# The chemosensory toolkit of the cursorial spider *Pisaura mirabilis*

**DOI:** 10.1038/s42003-025-09127-z

**Published:** 2025-11-29

**Authors:** Mohammad Belal Talukder, Carsten H. G. Müller, Andreas Fischer, Vedanti Mahimkar, Jonas O. Wolff, Gabriele B. Uhl

**Affiliations:** 1 General and Systematic Zoology, University of Greifswald, Greifswald, Germany; 2 Evolutionary Biomechanics, University of Greifswald, Greifswald, Germany

**Keywords:** Chemical ecology, Entomology, Animal physiology

## Abstract

Chemical sensing is essential for animals to locate food, avoid predators, and find mates. Like many arthropods, spiders rely on chemosensory inputs, but their toolkit remains largely unknown. Here, we investigate the basics of chemosensing in the cursorial spider *Pisaura mirabilis*. Using electron microscopy, we identified two types of chemosensory sensilla. Tip-pore sensilla occur on legs and pedipalps of both sexes, while wall-pore sensilla are found on walking legs of adult males only. Tip-pore sensilla are classified as contact chemosensilla, while wall-pore sensilla are classified as odor-detecting sensilla. Our behavioral studies confirm that males are attracted to female odor. The distribution of these sensilla types supports their functions: tip-pore sensilla occur mainly at the tips of the legs, whereas wall-pore sensilla occur closer to leg bases, not contacting the substrate. These findings expand our knowledge of chemosensing in spiders and have implications for research on arthropod chemical ecology.

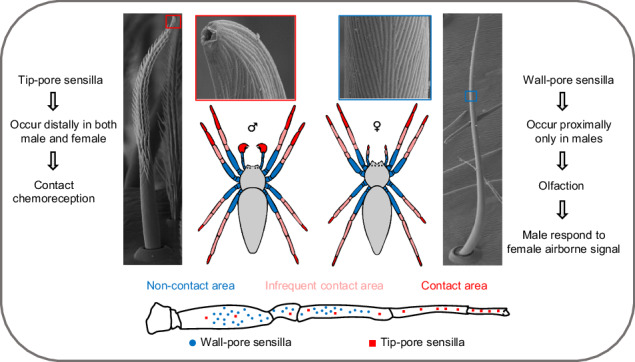

## Abstract (web summary)

Discovery of chemosensory sensilla with different distributions on the body appendages and between the sexes of a cursorial spider, combined with evidence of olfactory mate attraction, providing insights into chemical communication in spiders.

## Introduction

Chemical sensing is a crucial sensory modality across all animal phyla^1^, supporting a wide range of behaviors essential to ecological success and reproductive fitness. Land-living arthropods, such as insects, utilize highly specialized chemosensory systems to detect and interpret environmental chemical information from, e.g., food sources, predators, and conspecifics in the form of surface-bound compounds upon contact and airborne molecules. In insects, contact chemoreception (gustation/taste) is mediated by sensory hairs (sensilla) featuring a terminal or subterminal pore. These tip-pore sensilla are located on the mouthparts, antennae, ovipositors and distal segments (podomeres) of the legs^2^. In contrast, the perception of airborne chemicals (olfaction/smelling) is facilitated by sensilla with wall pores distributed along their shafts, which are predominantly found on the insect antennae^3^. In both types of sensilla, chemicals enter their pore(s) and bind to receptor cell dendrites within the sensillum shaft^4,5^. Similarly, in crustaceans, contact chemosensilla (gustatory sensilla) with a terminal pore are distributed unevenly across the body, with dense concentrations on mouthparts and walking legs. In contrast, the olfactory sensilla of crustaceans, known as aesthetascs, are thin-walled pegs that lack pores but contain numerous olfactory neurons and are located primarily on the first and second antennae^6^.

Compared to insects and crustaceans, limited knowledge exists regarding the chemical senses of other arthropods, limiting our comprehension of their sensory structures and the evolutionary history of these structures within this clade. For spiders, many behavioral studies support the use of both gustatory and olfactory information^7–9^. For example, males are attracted to female-produced airborne sex pheromones^10–13^, and upon contact, gustatory female sex pheromones elicit courtship behavior in males^12,14,15^. Other than in the mating context, there is behavioral evidence that spiders use contact chemoreception and olfaction for habitat selection^16,17^, prey detection^18,19^, and predator avoidance^20,21^.

The cuticular chemosensory structures currently identified in spiders are tip-pore sensilla^22–24^, which are found in large numbers on all walking legs and pedipalps of both sexes. These sensilla have been shown to function as contact chemoreceptors in an electrophysiological study on the spider *Cupiennius salei*^25^. Prior to recent findings, reports of wall-pore sensilla in spiders were lacking (but see ref. ^22^). Wall-pore sensilla have now been found in the orb-weaver spider *Argiope bruennichi*, where they occur in large numbers on all walking legs and exclusively in males^26^. Using single sensillum electrophysiological recordings, it was demonstrated that the male-specific wall-pore sensilla respond to the female-produced airborne sex pheromone^26^. Adult females release the pheromone from their webs and attract males from a distance^10^. As is typical for web-building spiders, *A. bruennichi* females are stationary in their webs, whereas adult males actively search for females. However, many spider species are cursorial and do not reside in silken webs; instead, they roam freely and deposit dragline silk as they walk. Behavioral observations have demonstrated that in cursorial spiders such as lycosids, males follow female silk trails to locate potential mating partners^27,28^. These findings suggest that cursorial spiders rely primarily on silk-bound chemicals (gustation) for mate finding (but see Searcy et al.^29^ for evidence of olfaction). An examination of the sensilla apparatus of a cursorial spider, in conjunction with a behavioral assessment of the ability to perform olfaction during mate search, will help contribute to the understanding of the chemosensory world of non-web-building spiders.

The European nursery web spider *Pisaura mirabilis* (Clerck, 1757), also known as the nuptial-gift-giving spider, is a compelling model for exploring chemical communication in cursorial spiders. The lifestyle of *P. mirabilis* combined with well-studied knowledge of its mating behavior^30–33^, which relies on silk-bound chemical information^34–37^, makes it particularly valuable for investigating chemosensory mechanisms. Furthermore, choice experiments have shown that *P. mirabilis* utilizes chemical information from plants for habitat selection^38^ and avoids the cuticular hydrocarbons of an ant predator^21^. However, most experiments have not differentiated between the sensory modes of gustation and olfaction. Although mating-related contact chemoreception (gustation) has been supported in several studies^35,39^, whether olfaction is a relevant sensory mode remains to be investigated. The recent identification of putative wall-pore sensilla in adult *P. mirabilis* that are male-specific^26^ suggests that olfaction plays a major role during mate search and possibly other contexts in this species.

To shed light on the chemical communication system of cursorial spiders, we investigated the chemosensory structures of *P. mirabilis* and hypothesized that *P. mirabilis* possesses diverse types of chemosensilla if they are capable of performing contact-chemoreception and olfaction. Using high-resolution electron microscopy, we examined legs and pedipalps for candidate sensilla in both sexes and subadults and characterized the morphology and distribution of these sensilla. To address the knowledge gap regarding olfaction, we tested the hypothesis that males detect and respond to female-emitted airborne signals.

## Results

### Tip-pore sensilla

#### External morphology of the tip-pore sensilla

**Fig. 1:**
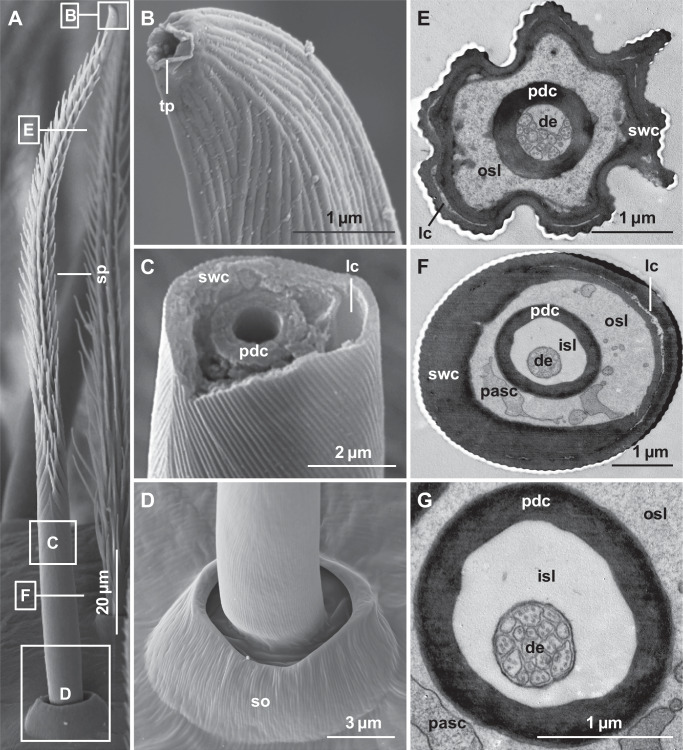
External morphology and internal anatomy of tip-pore sensilla in *Pisaura mirabilis.* **A**–**D** Scanning electron microscopy (SEM) and **E**–**G** transmission electron microscopy (TEM)**. A** An overview of a tip‒pore sensillum showing a spiny, tapered shaft (sp) with a blunt tip. Note that the basal region of the shaft is not spiny but finely ribbed. **B** Close-up image of the tip region, highlighting the terminal pore (tp). **C** A broken sensillum shaft revealing the hollow center surrounded by the cuticular peridendritic shaft cylinder (pdc). Note the longitudinal canal (lc) within the shaft wall cuticle (swc). **D** Round, elevated socket (so) of the sensillum with a nonporous, finely ribbed base. **E** Cross-section of the distal region of the sensillum shaft, showing a strongly corrugated, star-shaped cross profile of the shaft wall cuticle. A ring-like formation of thin, partly fused longitudinal canals (lc) is present within the shaft wall. The sensillum shaft contains two distinct lymph spaces, the inner (isl) and outer (osl) sensillum lymph spaces, which are separated by the thick peridendritic shaft cylinder. The inner lymph space houses 17 dendrites (de) of chemoreceptive cells. **F** Cross-section of the basal shaft region, showing a finely ribbed shaft wall cuticle that contains a ring-like canal system. The outer sensillum lymph space shows cytoplasmic projections of accessory sheath cells (pasc). **G** Close-up view of the inner sensillum lymph space (isl), surrounded by the thick peridendritic shaft cylinder. The inner sensillum lymph space contains 16 tightly packed chemoreceptive dendrites (de).

Sensilla with a blunt tip and a slightly oval terminal pore (Fig. 1A, B), measuring 0.4 to 0.7 µm in diameter (*N* = 10 pores for each sex), were found on all walking legs and pedipalps of both males and females of *Pisaura mirabilis*. They are hair-like, appear slightly S-shaped, and feature spirally oriented microtrichia (spine-like protuberances) along the shaft (Fig. 1A). The spination begins approximately 30 µm from the finely ribbed shaft base and extends up to 10–15 µm below the tip. The tip region contains many shallow longitudinal grooves (Fig. 1B, C). The shafts of the tip-pore sensilla emerge from a round, slightly elevated crater-like socket (Fig. 1D) and project from it at a steep angle (50–65°) to the leg surface cuticle, in contrast to the longer, branched, striated, and sharp-pointed tactile setae (mechanoreceptors). In females, the sensillum shafts are approximately 188.14 ± 40.80 µm [arithmetic mean ± standard deviation] long (*N* = 30), whereas in males, they are slightly shorter (175.15 ± 37.39 µm, *N* = 30), with no significant difference between the sexes (Student’s *t*-test, t = −1.19, *p* = 0.24). The shaft diameter is very similar between the sexes, with average values of 6.58 ± 0.71 µm (*N* = 30) in females and 6.24 ± 0.65 µm (*N* = 30) in males, which is not statistically different (Student’s *t*-test, t = −1.78, *p* = 0.082) (Supplementary Table 1).

#### Internal anatomy of the tip-pore sensilla

We examined the internal anatomy of the tip-pore sensilla from the femur of the 1st walking leg of both males and females of *P. mirabilis*. Ultrathin cross-sections of the middle region of the sensillum shaft reveal a star-shaped profile and a distinctly corrugated surface of the shaft wall cuticle (Fig. 1E), corresponding to the spirally oriented, ribbed microtrichia observed with scanning electron microscopy (SEM) (Fig. 1A), whereas in the proximal region, the shaft possesses a circular profile and a finely ribbed surface (Fig. 1C, D). The thick shaft wall cuticle features narrow lymph canals running longitudinally inside the shaft wall, resulting in a double-wall appearance in cross-section (Fig. 1C, E, F). None of the cross-sections examined revealed that these longitudinal shaft canals are connected to the lymph space or to the outside. Cross-sections show a hollow sensillum shaft filled with lymph (Fig. 1E, F). The lymph space is divided into two compartments. These compartments are separated by a thick cuticular tube called the peridendritic shaft cylinder (Fig. 1C, E–G) (according to Müller et al.^24^). The inner compartment, called the inner sensillum lymph space, contains 15–18 likely non-branching dendrites (Fig. 1E–G), which are likely chemoreceptive. Each dendrite is characterized by a set of microtubules. All dendrites running through the shaft terminate below the terminal pore. The outer compartment, called the outer sensillum lymph space, surrounds the inner compartment (Fig. 1E–G). The outer sensillum lymph space often contains cytoplasmic processes of accessory sheath cells located beneath the leg cuticle (Fig. 1F, G). Tubular bodies, indicative of  amechanoreceptive  function, typical for tip-pore sensilla in spiders, is present in the socket region of this sensillum. The somata of both chemoreceptive and mechanoreceptive neurons are located beneath the socket of the sensillum, as shown in *A. bruennichi*^24^.

#### Distribution of tip-pore sensilla

**Fig. 2:**
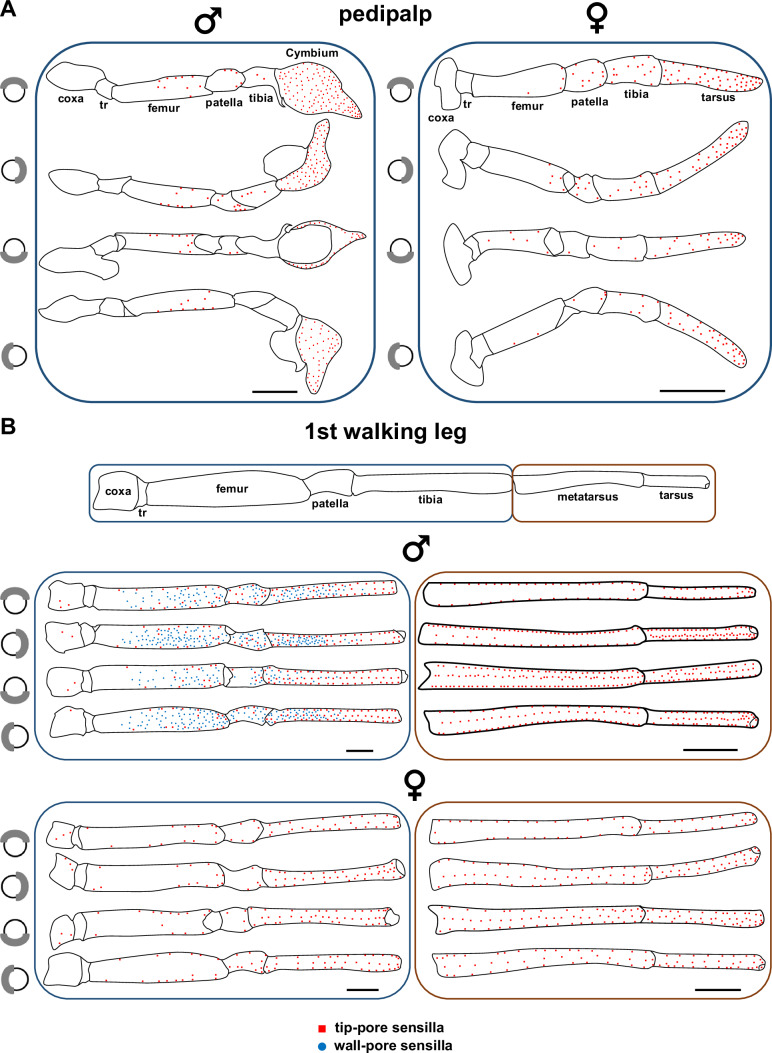
Distribution of chemosensilla in *Pisaura mirabilis.* **A** Pedipalp of a male (♂) and a female (♀). **B** First walking leg of a male (♂) and a female (♀). Red squares represent tip-pore sensilla and blue circles wall-pore sensilla. Each panel shows four perspectives from top to bottom: dorsal, prolateral, ventral, and retrolateral. Notably, wall-pore sensilla are present only on the walking leg of males. tr: trochanter. All scale bars: 1 mm.

In *P. mirabilis*, adult males possess approximately 4100 tip-pore sensilla distributed across all walking legs and pedipalps, whereas females have approximately 2900 (Fig. 2, Supplementary Figs. 1, 2A–C). On all walking legs of both males and females, these sensilla are most abundant on the tarsus, metatarsus, and tibia; occur in lower numbers on the patella and femur; and are absent on the trochanter (Fig. 2B, Supplementary Fig. 1). In both sexes, there are few (3–9) tip-pore sensilla on the coxa of the walking legs. On the tarsus, metatarsus, and tibia of all walking legs in both males and females, the tip-pore sensilla are arranged in seven to eight rows along the segment axes. In males, the number of tip-pore sensilla on the first and second walking legs was 43–67% greater than on the third and fourth pairs. Similarly, in females, the first and second walking legs bore 29–41% more tip-pore sensilla than the posterior leg pairs (Supplementary Fig. 2C). On all walking legs, tip-pore sensilla were predominantly distributed along the lateral and ventral surfaces and comparably rare on the dorsal sides (Fig. 2B, Supplementary Fig. 1). On the pedipalps of both sexes, tip-pore sensilla occurred mainly on the distal segment, specifically, on the tarsus in females and the cymbium (the outer part of the modified palpal tarsus) in males. In both sexes, the number of tip-pore sensilla on the tarsus and cymbium of the pedipalps was two- to four-fold higher than the combined total found on the proximal segments (femur, patella, and tibia) (Fig. 2A). Toward the body, no tip-pore sensilla were observed on the trochanter or coxa of the pedipalps in either sex.

### Wall-pore sensilla

#### External morphology of wall-pore sensilla

**Fig. 3:**
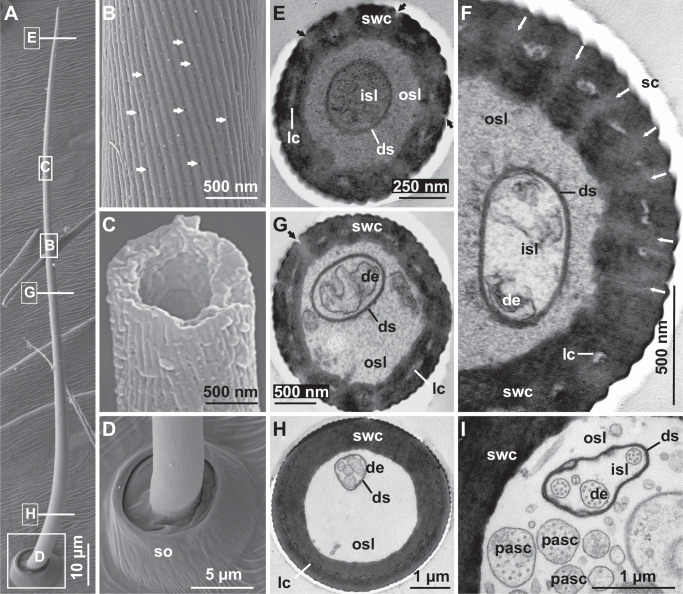
External morphology and internal anatomy of wall-pore sensilla in *Pisaura mirabilis.* **A**–**D** SEM and **E**–**I** TEM. **A** An overview of a wall-pore sensillum with a curved-tapered shaft and round socket. **B** Close-up image of the middle region of the sensillum shaft showing a grooved and ridged surface with multiple pore-like depressions (some are indicated by white arrows) located within the grooves. **C** A broken shaft revealing the hollow center (lymph space) of the sensillum. **D** Socket (so) of the sensillum with a nonporous, finely ribbed base. **E** Cross-section of the tip region of the sensillum shaft showing wall pores (black arrows) in the shaft wall cuticle (swc). The outer lymph space (osl) and inner lymph space (isl) are separated by a dendritic sheath (ds).  Dendrites are not clearly discernible. Longitudinal canals (lc) are present in the shaft wall cuticle. **F** Spoke canals (sc) (marked with white arrows) in the shaft wall cuticle connect the outer environment to the lymph spaces. Some partially disintegrated dendrites (de) of receptor cells are visible in the inner lymph space. A longitudinal canal is present in the shaft wall cuticle. **G** Cross-section of the mid-region of the sensillum shaft, showing pores connected to the lymph space via spoke canals. Two chemoreceptive dendrites (de) are visible in the inner lymph space. **H** Cross-section of the basal region of the sensillum shaft showing four dendrites (de) enclosed by the dendritic sheath. The longitudinal canals are mostly fused and form a compact ring-like structure. **I** Close-up of the basal region of the sensillum shaft in cross-section, highlighting four dendrites in the inner lymph space. The outer lymph space contains cytoplasmic projections of accessory sheath cells (pasc).

The wall-pore sensilla are thin, trichoid hairs with round, slightly elevated crater-like sockets (Fig. 3A–D). These sensilla occur exclusively on all walking legs of adult males and are not found in females or subadult males (Supplementary Fig. 3). The sensilla have a curved shape with a tapered tip. The insertion angles of the wall-pore sensilla are slightly steeper than those of the mechanoreceptive sensilla (tactile hairs) found on all leg and pedipalp segments in high abundance. On average, the wall-pore sensilla of *P. mirabilis* are 141.78 ± 11.41 µm long (*N* = 15) (Supplementary Table 2). The sensillum diameter is 2.12 ± 0.16 µm, as measured approximately 20 µm distal to the hair base (*N* = 15). The surface of the sensillum shaft shows fine diagonal ridges and grooves (Fig. 3B), which are longitudinally oriented toward the tip. Numerous pore-like invaginations (Fig. 3B), with an average diameter of 41.93 ± 3.26 nm (*N* = 45), are distributed inside the grooves over almost the entire length of the shaft, except for the sensillum base region (Fig. 3D). No marked differences in size or shape were observed in the wall-pore sensilla across different walking legs and their segments in male *P. mirabilis* (Supplementary Table 2).

#### Internal anatomy of wall-pore sensilla

For transmission electron microscopy (TEM) analysis, we focused on wall-pore sensilla located on the femur of the 1st walking leg of male *P. mirabilis*. Cross-sections taken from different regions of the shaft reveal that the wall-pore sensilla possess two lymph spaces (Fig. 3E–I). The outer lymph space surrounds the inner sensillum lymph space that is lined by a dendritic sheath (Fig. 3E–I). The inner lymph space hosts 2–4 (mostly 4) dendrites characterized by a set of microtubules (Fig. 3F–I). The dendrites are tightly packed inside the dendritic sheath, which is present throughout the shaft. The analyses of serial ultrathin cross-sections revealed that there are pores in the shaft cuticle (Fig. 3E, G). These pores are connected to thin canal-like structures (radial spoke canals, approximately 100 nm in diameter; Fig. 3F) that traverse the shaft wall cuticle and open into the outer sensillum lymph space. As these spoke canals run obliquely through the shaft wall, we did not capture their full course in our cross-sections. Pore tubules, such as those found in single-walled sensilla of ticks (*Amblyomma variegatum*)^40^, were not observed in the spoke canals of the wall-pore sensilla of *P. mirabilis*. The shaft wall cuticle exhibits many narrow and distinct longitudinal lymph canals in a ring-like formation, giving the shaft wall a double-walled appearance (Fig. 3E–H). At the basal shaft level, most of the longitudinal shaft canals fuse to form a ring (Fig. 3G). The longitudinal shaft canals do not interconnect with the radial spoke canals. In some sections, the outer sensillum lymph space contains cytoplasmic extensions of the accessory sheath cells (Fig. 3I) that are located beneath the leg cuticle. Like in the wall-pore sensilla of *A. bruennichi*^26^, we did not detect tubular bodies, key components of mechanoreceptors, in the socket region, indicating that these sensilla are not multimodal (chemo-and mechanosensing), as is characteristic of tip-pore sensilla.

#### Distribution of male-specific wall-pore sensilla

The male-specific wall-pore sensilla of *P. mirabilis* are abundant (approx. 3000 sensilla) and somewhat irregularly arranged on the proximal leg segments (femur, patella, and tibia) of all walking legs of the males (Fig. 2B, Supplementary Figs. 1, 4, 5). They are most abundant in the middle to distal regions of the femur and patella and in the proximal region of the tibia in the first, second, and fourth leg pairs. In contrast, wall-pore sensilla were not found on the distal segments (metatarsus and tarsus) of any leg or on the male pedipalps. Compared with the dorsal and ventral sides, the wall-pore sensilla are more densely distributed along the lateral sides of the legs (Fig. 2B, Supplementary Fig. 1). The first and second legs each possess approximately 460–470 wall-pore sensilla, whereas the fourth leg possesses approximately 340, and the shorter third leg has approximately 200 wall-pore sensilla (Supplementary Fig. 2D).

A comparative summary of the structural characteristics and distribution of tip-pore and wall-pore sensilla, including cross-references to the corresponding figures, is presented in Table 1.

**Table 1:** Comparative summary of external morphology, internal anatomy, and distribution of tip-pore and wall-pore sensilla in *Pisaura mirabilis*

	Feature	Tip-pore sensilla	Wall-pore sensilla
Shaft and socket external morphology	Occurrence	Both sexes; on all walking legs and pedipalps	Adult males only; on all walking legs
	Shape	Slightly S-shaped; blunt tip (Fig. 1A)	Curved with tapered tip (Fig. 3A)
	Shaft surface	Finely vertically ribbed shaft base (Fig. 1C, D), strongly ribbed medially and distally, ribs carry spirally arranged microtrichia (Fig. 1A); shallow longitudinal grooves at tip (Fig. 1B)	Finely vertically ribbed shaft base (Fig. 3C, D), fine diagonal ridges medially and distally (Fig. 3B); longitudinal grooves with numerous wall pores (Fig. 3B)
	Pore type and location	Single oval pore (400–700 nm diameter) at tip	Numerous wall pores (~42 nm diameter) along the shaft, except at the base
	Socket	Circular, slightly elevated crater-like socket (Fig. 1D)	Circular, slightly elevated crater-like socket (Fig. 3D)
	Average length	♂: ~175 µm; ♀: ~188 µm	♂:~142 µm
	Average diameter	♂: ~6 µm, ♀: ~6.5 µm;	♂:~2 µm
Shaft and socket internal anatomy	Location examined	Femur of 1st walking leg (males and females) (Fig. 2B)	Femur of 1st walking leg (males only) (Fig. 2B)
	Shaft cross-profile	Star-shaped (mid-shaft, Fig. 1E); circular (base, Fig. 1F)	Circular; finely corrugated (Fig. 3E–G)
	Shaft wall features	Double-walled appearance with longitudinal shaft canals (e.g., Fig. 1E)	Double-walled appearance with longitudinal shaft canals (Fig. 3F, G)
	Longitudinal shaft canals	Present; partly coherent (Fig. 1E-F), connected with the outer sensillum lymph space at the shaft base	Present; dispersed (Fig. 3E, G), connected to outer sensillum lymph space at shaft base; not connected with radial spoke canals (Fig. 3F)
	Sensillum lumen	Hollow (Fig. 1C), filled with lymph; separated into inner and outer sensillum lymph spaces (e.g., Fig. 1F)	Hollow, filled with lymph; separated into inner and outer sensillum lymph spaces (Fig. 3F)
	Outer lymph space	Present; surrounds inner lymph space; contains sheath cell processes (Fig. 1F)	Present; surrounds inner lymph space; contains sheath cell processes (Fig. 3I)
	Distal processes of accessory sheath cells	Present in the outer sensillum lymph space (Fig. 1F); continuing to the tip region	Present in the outer sensillum lymph space (Fig. 3I); continuing to the tip region
	Inner lymph space	Present; enclosed by a continuous dendritic sheath (proximal shaft region, Fig. 1G) and peridendritic shaft cylinder (median and distal shaft region, Fig. 1F)	Present; enclosed by a continuous dendritic sheath (Fig. 3E–I)
	Number of dendrites	15–18 dendrites (Fig. 1E, G)	2–4 (mostly 4, Fig. 3I) dendrites
	Spoke canals (pore canals)	Not present	Present; connect wall pores with outer sensillum lymph space; no contact with longitudinal canals (Fig. 1F), ~100 nm diameter
	Mechanoreceptive structures	Present; 2 tubular bodies at the socket region (multimodal sensillum)	Absent; no tubular bodies; not multimodal
Distribution	Total number	♂: ~4100; ♀: ~2900 sensilla	♂: ~3000 sensilla
	Leg segments with sensilla	All segments except trochanter (Fig. 2B)	Only on segments: femur, patella, tibia (Fig. 2B)
	Most abundant on	Tarsus, metatarsus, tibia (Fig. 2B)	Femur, patella, tibia (Fig. 2B)
	On pedipalps	Present mostly on distal segments (Fig. 2A)	Absent
	On walking legs	Present on all legs; arranged in 7–8 rows, mostly on lateral and ventral surfaces (Fig. 2B)	Present on all legs; irregularly arranged; mostly on lateral surfaces (Fig. 2B)

### Olfactometer test

**Fig. 4:**
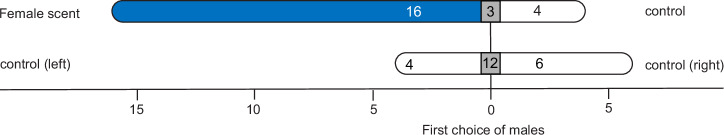
Responses of male *Pisaura mirabilis* to the scent of females or stimulus-free controls. The numbers on the blue and white bars represent responders, whereas the central gray boxes represent non-responders (males who did not leave the holding tube).

Olfactory attraction of males to females was observed (binomial test, *p* = 0.012; Fig. 4), with 16 (70%) out of 23 males choosing the branch that contained a female, four (17%) choosing the control branch, and three males (13%) not leaving the holding tube. In the control experiment, no side preference was observed, as four males (18%) chose the left branch, six males (27%) chose the right branch (binomial test, *p* = 0.754; Fig. 4), 12 males (55%) exhibited no behavioral response (Fig. 4), and one male died prior to their second trial. Males were more likely to make a choice when exposed to a female scent (87%) than when they were not exposed to a female scent (45%) (Fisher’s exact test, *p* = 0.005; Fig. 4).

### Contact area versus non-contact area of body appendages

**Fig. 5:**
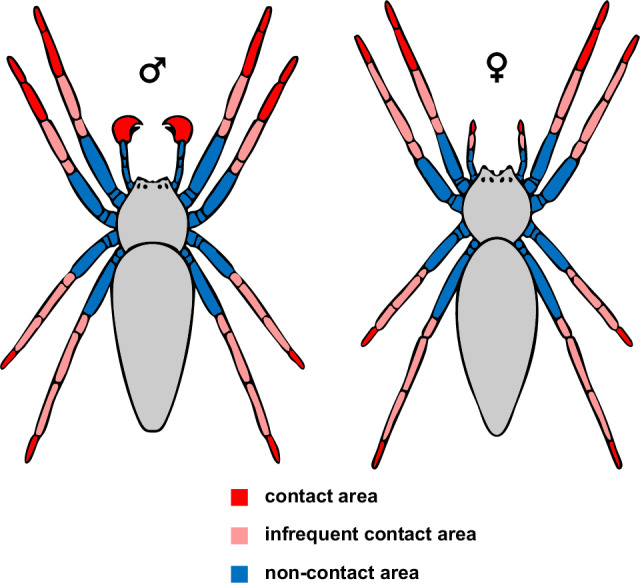
Schematic representation of contact and non-contact areas of walking legs and pedipalps in *Pisaura mirabilis* males (♂) and females (♀). Contact and non-contact areas were categorized on the basis of substrate contact during locomotion, prey capture, and interactions with mating partners via high-speed video recordings. Segments with a contact probability of more than 50% across all trials were classified as contact areas (Red). Segments with a contact probability of 1% to less than 50% were categorized as infrequent contact areas (Rose). The segments that never contacted the substrate during the trials were classified as non-contact areas (Blue). The segments from tip to body are the tarsus, metatarsus, tibia, patella, femur, trochanter, and coxa.

We conducted a high-speed (500 frames per second) video analysis to define contact and non-contact regions of the body appendages of male and female *P. mirabilis* during locomotion (Supplementary Movie 1), on various surfaces and during silk probing, prey capture (Supplementary Movie 2), and mating (Supplementary Movie 3). The contact and non-contact areas of the walking legs and pedipalps of *P. mirabilis* were delineated on the basis of the contact probability across all pooled contexts: locomotion on four different substrates (*N* = 6 spiders × 4 substrates = 24 trials per sex), female’s silk probing by males (*N* = 6 trials with 6 males), prey (smaller housefly and larger cricket) capture (*N* = 6 spiders × 2 prey types = 12 trials per sex), and mating (*N* = 6 male–female pairs) (Fig. 5, Supplementary Tables 3, 4). Upon pooling all contexts (Supplementary Table 4), we observed that the ventral and lateral sides of the tarsus of all walking legs exhibited frequent substrate contact in both sexes (55–100%). The metatarsus in males demonstrated frequent substrate contact on the first two walking legs (52–53%), whereas in females, frequent contact was observed solely for the metatarsus of the first walking legs (53%). In both sexes, the tibia‒patella segments of all walking legs exhibited infrequent substrate contact across all contexts (1–11%), and the femur did not establish contact with the substrate or mating partner in any context of our experimental trials. With respect to pedipalps, the cymbium in males and the tarsus in females frequently probed substrates, nuptial gifts, and prey, with contact frequencies ranging from 86 to 100%. Conversely, the remaining pedipalp segments in both sexes rarely established contact in any context. Detailed observations of all the behavioral contexts are provided in the supplementary information (Supplementary Note 1, Supplementary Tables 3, 4).

## Discussion

We detected two distinct types of chemosensory sensilla in the cursorial spider *P. mirabilis*: tip-pore sensilla and wall-pore sensilla. The first type, tip-pore sensilla (Fig. 1), is associated with contact chemoreception (gustation), a well-documented function in insects^5^, crustaceans^6^, and one spider species^25^. In *P. mirabilis*, tip-pore sensilla are present in both sexes, as well as in subadults, and are distributed across all walking legs and pedipalps (Fig. 2). They are predominantly located on the distal segments of the appendages, which frequently contact substrates (Fig. 5), suggesting their role in detecting surface-bound chemical information. The second type, wall-pore sensilla (Fig. 3), functions as olfactory sensilla, a role demonstrated in insects^4^, crustaceans^6^, and more recently in a spider^26^. Our Y-tube olfactometer assays demonstrated that *P. mirabilis* males respond to airborne signals emitted by females (Fig. 4). In *P. mirabilis*, wall-pore sensilla are exclusive to adult males and absent in females and subadult males (Supplementary Fig. 3). These sensilla are located on the proximal segments of all walking legs (Fig. 2B, Supplementary Fig. 1), areas that rarely contact substrate, prey, or mating partners (Fig. 5), which is consistent with findings in the orb-weaving spider *A. bruennichi*^26^, corroborating their specialization for detecting airborne signals emitted by females.

Tip-pore sensilla are present on all walking legs and pedipalps of both sexes and subadult *P. mirabilis*. These sensilla feature chemoreceptive dendrites housed within a trichoid shaft, with the dendrites terminating at a blunt terminal pore (Fig. 1). This pore provides the only connection between the dendrites and the external environment, supporting the role of tip-pore sensilla in contact chemoreception, as confirmed in, e.g., insects^5^, crustaceans^6^, and arachnids such as opilionids and ricinulei^41,42^. The gustatory role of tip-pore sensilla has also been demonstrated in the cursorial spider *Cupiennius salei* through electrophysiological studies^25^. Although the outer morphology of the tip-pore sensilla can differ between species, the internal ultrastructure of the sensilla shafts in *P. mirabilis* and *C. salei* is highly similar to those of other cursorial spiders and web-building spiders^24,43^. In all cases, tip-pore sensilla also entail a mechanoreceptive component^24,25,43^.

In *P. mirabilis*, tip-pore sensilla are characterised by steeper angles (Supplementary Table 1) than pure mechanoreceptive sensilla and are more densely distributed on the distal segments (tarsus and metatarsus) of the walking legs and pedipalps, with fewer sensilla on the proximal segments (tibia, patella, and femur) (Fig. 2, Supplementary Fig. 1). A similar distal abundance of tip-pore sensilla with steeper angles has been observed in both web-building^23^ and cursorial spiders^43^. In various insect orders, the presence of tip-pore sensilla on the mouthparts and distal segments of the walking legs is common^2^. For example, in leaf beetles (Coleoptera), the tip-pore sensilla present on the tarsi of the legs play a crucial role in host selection^44^. The distal placement and steeper insertion angles of tip-pore sensilla increase the probability of ontacting substrate-bound chemical information, facilitating gustation during navigation and during mating. Our video observations further support this, demonstrating that distal segments of the body appendages of *P. mirabilis* frequently contact substrates during locomotion, silk probing, prey capture, and mating, whereas proximal segments rarely do (Fig. 5, Supplementary Tables 3, 4). Additionally, there are fewer tip-pore sensilla on the dorsal sides of all walking legs, where contact with the substrate is rare (Fig. 2B, Supplementary Fig. 1), which further corroborates the functional role of the tip-pore sensilla as gustatory organs.

There is a marked difference between the sexes in the number of tip-pore sensilla in *P. mirabilis*, with males possessing approximately 4100 sensilla compared to 2900 in females (Supplementary Fig. 4), despite their similar body sizes. In males, the highest densities occur on the distal segments of the first and second walking legs and on the cymbium of the pedipalps. When encountering the dragline silk of a female, males touch the silk with these segments, and their tip-pore sensilla likely play a role in the detection of the reproductive status of a female and affect their trail-following decisions^34,36,39,45^. Consequently, mate detection and assessment likely require a greater number of tip-pore sensilla in males. In more ancient spiders, such as liphistiids, gustation-based mate search is likely also accomplished through the available tip-pore sensilla^46^, but male-specific scopulate hairs have also been suggested to perform this function^47^. Male-specific gustatory sensilla are also known from the lady beetle *Semiadalia undecimnotata* (Coleoptera), where they occur on the antennae and have been proposed to play a role in mating-related functions^48^. The greater quantity of tip-pore sensilla in the cursorial spider *P. mirabilis* than in the orb-weaver *A. bruennichi*, which has 1000 sensilla in males and 2000 in females^23^, likely reflects the necessity for cursorial spiders to perceive more detailed information on prey presence, habitat quality, and predation risk while roaming in the undergrowth of the vegetation. In contrast, most stationary web-building spiders predominantly remain on their webs — an extended perceptual system through which they can even identify prey types^49^. In the web, physical contact occurs solely with intercepted prey items (with the exception of contact with a mating partner), which spiders examine with their gustatory sensilla prior to wrapping or during prey consumption^50^.

Wall-pore sensilla are found exclusively in adult male *P. mirabilis*. These sensilla possess multiple pores connecting to a central lymph space within the sensillum shaft via radial spoke canals, forming a pathway from the external environment to the chemoreceptive dendrites in the lymph space (Fig. 3). The presence of longitudinal shaft wall canals indicates that these multiporous sensilla belong to the class of double-walled sensilla, which are known to occur in insects, myriapods, and arachnids other than spiders. However, the wall-pore sensilla of *P. mirabilis* possess canals that lack slit-like grooves on the shaft surface and vase-shaped chambers below the pore, as in other arachnids^40^. Distal processes of accessory sheath cells within the shaft (Fig. 3I) have been observed in single-walled sensilla with pore openings^40^, similar to those found on the tips of the sensory legs of amblypygid spiders^22^. The wall-pore sensilla of male *P. mirabilis* closely resemble those of *A. bruennichi*, for which the perception of the airborne female sex pheromone was corroborated through electrophysiological tests^26^. The wall-pore sensilla of both species, however, differ in ultrastructure from the single wall-pore sensillum described for a gradungulid species, a basally branching spider taxon^22^. Consequently, whether wall-pore sensilla evolved once within spiders or several times convergently remains to be investigated.

*P. mirabilis* males possess approximately 3000 wall-pore sensilla on all walking legs, predominantly in the proximal leg segments (Fig. 2B, Supplementary Fig. 1), which are non-contact areas (Fig. 5). This distribution suggests that their ability is to detect airborne signals released by females, similar to *A. bruennichi* males^26^*. Argiope bruennichi* males possess approximately 7000 male-specific wall-pore sensilla, which are likewise concentrated in non-contact areas of their walking legs and are finely tuned to airborne female-produced sex pheromone^26^. The greater number of wall-pore sensilla in *A. bruennichi* males may indicate a greater reliance on olfaction for mate search. Male *A. bruennichi* are attracted from substantial distances to stationary females by signals released from the web^10^, whereas *P. mirabilis* males may utilize both olfactory and gustatory information from female dragline silk in their environment.

In arthropods, male-specific olfactory sensilla are abundant and typically specialize in detecting sex pheromones. For example, in a moth species, males possess approximately 50,000 specialized antennal trichoid sensilla that are dedicated to detecting female sex pheromones, whereas other olfactory sensilla in both males and females respond to general odors, as reviewed previously^4,51^. Male-specific olfactory sensilla have also been found in other moth species^52–54^. Similarly, in aquatic mysids (crustaceans), approximately 2000 male-specific olfactory sensilla located basally on the first antennae have been proposed to function as pheromone receptors^55^. These observations support the hypothesis that male-specific wall-pore sensilla in *P. mirabilis* likewise have evolved to enhance the detection of airborne female sex pheromones substances.

Our behavioral assays indeed demonstrated that *P. mirabilis* males respond to airborne signals emitted by females (Fig. 4). This finding aligns with observations in the lycosid spider *Pardosa milvina*, where males are attracted to airborne signals from adult virgin females but not from subadult females or males^29^. The sexual pheromone of *P. mirabilis* responsible of eliciting remote attraction in our behavioral trials remains unidentified. We could not detect any pheromone candidate via gas chromatography‒mass spectrometry (GC‒MS), possibly because these signals involve polar compounds that are undetectable via GC‒MS^7^. Recently, liquid chromatography coupled with tandem mass spectrometry was used to identify polar olfactory and gustatory sex pheromones of widow spiders^11,12,56^, which have been elusive in GC‒MS analyses. Similar investigations for *P. mirabilis* are being conducted to identify the compounds responsible for mate attraction in a cursorial spider.

The olfactory capabilities of subadult and female spiders remain uncertain, as wall-pore sensilla have been found exclusively in male spiders^26^. Nevertheless, studies indicate that female and juvenile cursorial spiders utilize olfactory information for purposes such as habitat selection^16^ and prey detection^19^. Consequently, it can be postulated that tip-pore sensilla potentially serve dual gustatory (taste) and olfactory (smell) functions, analogous to certain tip-pore chemoreceptors identified in insects^5,57,58^. Apart from this dual-function hypothesis, a functional-zone hypothesis warrants investigation to determine whether the tip-pore sensilla located on the proximal segments of the legs, where direct contact with surfaces is infrequent, are involved in olfaction, and those in the distal part of the leg are limited to gustation. Consequently, the function of the tip-pore sensilla may vary depending on their location, as has been observed in insects^59^.

We set out to explore the chemosensory toolkit of arthropods other than insects as a step to unravel the evolution of chemosensing within arthropods. We elucidated the chemosensory apparatus and mate-finding mode of the cursorial spider *Pisaura mirabilis*. Tip-pore sensilla, present in both sexes and associated with contact chemoreception, and male-specific wall-pore sensilla linked to olfaction were discovered and described on the basis of their outer and inner anatomical features. The role of olfaction in mate search was demonstrated by male attraction to female scents. Morphological and behavioral findings suggest that male-specific wall-pore sensilla function in female pheromone detection, as observed in insects and crustaceans. Male *P. mirabilis* possess wall-pore sensilla on all walking legs in regions close to the body that normally do not contact the substrate, which corroborates their function in olfaction. The absence of wall-pore sensilla in females and immature male spiders raises questions regarding the sensilla involved in olfaction other than mate search. Candidates are tip-pore sensilla that may form functional zones depending on their location on the legs. More broadly, this study contributes to our understanding of the ecological and evolutionary drivers of chemosensory diversity in arthropods, offering insights into how organisms detect and respond to chemical information in their environment and providing material for comparative analyses of sensory organs and their evolution across arthropods.

## Methods

### Study species

Juvenile, subadult (one molt prior to adulthood), and adult *Pisaura mirabilis* (Clerck, 1757) individuals of both sexes were collected each year from 2021 to 2024 from grasslands in Greifswald, Germany (54° 05’ 49.91“N 13° 23’ 16.58”E), and brought to the laboratory. The spiders were individually housed in *Drosophila* culture tubes measuring 5 cm in diameter and 10 cm in height, with a netted top and a sponge lid at the bottom^35^. Inside each vial, a substrate of artificial aquarium plants was provided. To maintain high humidity levels, vials with sponges at the bottom were placed in trays filled with water. The spiders were kept at a constant temperature of 22 °C (±5 °C) under a 12:12 h light‒ dark cycle. Adult and subadult spiders were fed one housefly (*Musca domestica*) or blowfly (*Lucilia caesar*) twice a week, whereas juveniles were provided with several fruit flies (*Drosophila hydei*) on the same schedule. Juveniles and subadults were monitored regularly for the presence of exuviae to monitor their development.

### Scanning electron microscopy: investigation of the outer morphology and mapping of chemosensilla

To investigate and map chemosensilla, freshly molted adult males and females (four from each sex), as well as four subadults of *P. mirabilis*, were anesthetized with CO_2_. They were then mounted on a polyethylene board (Plastazote) with their legs straightened and secured with wire clamps. The mounted samples were submerged and preserved in 80% ethanol. For SEM analysis, the straightened walking legs and pedipalps on the right side were detached and dehydrated through a graded ethanol series up to 99% and then dried using a Leica EM CPD300 critical point dryer. The straightened legs and pedipalps were vertically mounted on metal SEM stubs. The samples were then coated with gold-palladium for 2.5 min using a Polaron SC7640 sputter coater (Fisons Instruments). Then, the stubs were placed horizontally into a custom-made stub holder that allowed the samples to be rotated at various angles.

The samples were examined using a Zeiss EVO LS 10 SEM operated at 10 kV. The mounted samples were oriented perpendicular to the electron beam and rotated at 0°, 90°, 180°, and 270° to capture images from the dorsal, prolateral, ventral, and retrolateral sides of the body appendages. Owing to variation in the diameter of the segments of the body appendages (coxa to tarsus), we used a range of magnifications (×130 to ×220), but each segment was imaged at a consistent magnification for all males and females. The images were stitched together to reconstruct the relative positions of the sensilla on outline drawings of the spider’s legs, derived from multifocus stereomicroscopic photographs (Zeiss SteREO Discovery V20). Sensilla near the drawings´ borders appear on several maps but were excluded from the total count of sensilla. During mapping, the sensilla account for individual variation, and three males and three females were examined. All the sensillum parameters (length, diameter, insertion angle, and pore diameter) were measured via SEM built-in software. For a higher resolution of the properties of the sensillum shafts, a Zeiss SUPRA 40VP field emission SEM was used. The samples were cleaned with a 5% KOH solution and glacial acetic acid, following the methods described by Schneeberg et al.^60^, before they were subjected to critical point drying. The legs were then mounted onto SEM stubs and sputter-coated with an Au-Pd (80:20) mixture at 5 mA (Q150T ES, QUORUM, UK), resulting in a coating thickness of 10 nm.

### Transmission electron microscopy: investigation of the internal anatomy of chemosensilla

To prepare the samples for TEM, freshly molted samples—two males and two females of *P. mirabilis*—were anesthetized with CO₂. The walking legs were then dissected using micro-scissors while immersed in an ice-cold prefixative solution modified from Karnovsky’s protocol^61^, consisting of 2.0% glutaraldehyde, 2.5% paraformaldehyde, and 1.5% sucrose in 0.1 mol/L sodium phosphate buffer at pH 7.4. To improve tissue preservation, each leg segment was cut into 2–5 pieces, each measuring 1–3 mm in length, and incubated in the same prefixative solution. To further enhance fixation, the samples were subjected to three sets of 2 min microwave pulses at a power of 120 W using a PELCO BioWave Pro instrument equipped with a PELCO SteadyTemp Pro Thermo Cube for solid-state cooling. Throughout the BioWave application, the sample temperature was monitored, remaining between 8–10 °C prior to treatment and 9–15 °C afterward. The leg pieces were stored in the same prefixative solution at 4 °C for at least one night. The samples were then washed three times in 0.1 M sodium phosphate buffer for 45 min in total. The samples were subsequently postfixed with 2% osmium tetroxide for 3–4 h at room temperature (RT) and then washed three times with ddH₂O for a total of 30 min. The samples were dehydrated through a series of ethanol solutions (50%, 60%, 70%, 80%, 90%, 100%), with dehydration at each concentration up to 90% carried out for 2 × 10 min at RT. For the 100% ethanol stage, the samples were washed over 3 × 10 min. Subsequent infiltration of the samples with epoxy resin (Embed812, an Epon substitute) was conducted at RT using the solvent propylene oxide (PO) over several stages of dilution: 2 × 100% PO for 15 min in total, 66% for 2 h, 50% for 12 h, and 33% for 24 h. The samples were placed on a shaker throughout infiltration to support efficient infiltration. For pre-embedding, the samples were incubated in 100% epoxy resin and again placed on a shaker for 2 h. To further increase infiltration speed and efficiency, the samples were then transferred to a Heraeus VT-6025 vacuum heating cabinet for 2 h at 40 °C. During this time span, the samples were exposed to high vacuum conditions (3 × 30 min at 150 mbar, each phase separated by 10-min breaks at normal atmospheric pressure). For embedding, the samples were placed in silicon molds containing fresh epoxy resin. Each piece of the femur, tibia, patella, metatarsus, or tarsus was oriented in specific resin blocks: transverse, oblique, or longitudinal to the block face. The resin blocks were polymerized in a heating cabinet at 60 °C for 48 h. Transverse, horizontal, and longitudinal ultrathin sections (70–90 nm thick) of the tibia and femur were produced using a Leica UCT ultramicrotome. In areas from which ultrathin sections were obtained (with trimming gaps of 2–5 µm), semithin sections (700 nm thick) were also taken and stained with toluidine blue (dissolved in 1% sodium tetraborate) for examination under a light microscope. The ultrathin sections were placed on Formvar-coated slot grids (PLANO), stained with uranyl acetate and lead citrate for 4 min each, and then examined with a JEOL JEM-1011 transmission electron microscope operated at 80 kV. Digital photomicrographs were captured using a mid-mount camera (MegaView III, Soft Imaging System) with iTEM imaging software (Olympus, Soft Imaging Solution).

### Olfactometer test

Olfactory attraction of males to unmated female *P. mirabilis* was tested in December 2024 using Y-tube olfactometers (Supplementary Fig. 6) following established protocols^62^. Females were placed in stimulus chambers (26 × 2.5 cm glass tube with mesh on both sides) for 2 h to allow for acclimatization and silk deposition. The stimuli chambers were enclosed by oversized black mesh, where the females would eventually settle and remain visually occluded. The stimulus chamber was then connected by tubing to one arm of the Y-olfactometer (main stem: 24 cm long, side arms: 21 cm long, diameter: 2.5 cm), while a control chamber, identical but without a female, was connected to the other arm. To initiate the assay, a male was placed into a glass tube (26 × 2.5 cm, with mesh on both sides) for 2 min to acclimatize, while a pump drew 0.4 L/min air. The acclimatization tube was then connected to the stem of the Y-tube. The male was subsequently given 15 min to reach the end of either arm, which was considered a choice. Upon the first choice, the experiment was ended. To provide a grip for the spiders, all glass tubes held bamboo skewers (20 × 0.4 cm, Exnima, Damiel, Spain). Potential vibrational and visual female signals were blocked for the males by placing the setup on Styrofoam and interconnecting the glass chambers with flexible tubing and a rubber stopper with a hole. Tissue paper, with a hole, lined the three terminal stoppers connected to the tubing. The presentation sides were altered 50:50 across replicates. Bamboo skewers and tissues were discarded upon use, while the glassware was rinsed with water and baked for 2 h at 250 °C to remove any stimuli. The experiment was replicated 23 times with randomly paired males and females (*N* = 23 each). To test for any side bias, each male was tested again one week later in the same setup but without any female stimuli. Male olfactory choice data were statistically compared against random distribution using binomial or Fisher’s exact tests in R^63^.

### Contact and non-contact areas of body appendages

To identify the contact and non-contact areas on the appendages (legs and pedipalps) of male and female *P. mirabilis*, we recorded their leg and pedipalp movements using a high-speed camera (Miro LC320S, AMETEK Vision Research) at 500 frames per second (fps). Each recording was started immediately at the first contact with the given substrate and recorded for up to 8 s. The following behavioral scenarios were recorded under laboratory conditions: (a) locomotion, (b) silk probing by males, (c) prey capture, and (d) mating. For the locomotion trials, six males and six females of *P. mirabilis* were tested on four different substrate types (*N* = 6 × 4 = 24 trials per sex). These substrates were selected to represent surfaces commonly encountered by spiders in their natural habitat and included: a flat surface, fresh common nettles (*Urtica dioica*; on which we frequently find the species in the field), a horizontally positioned wooden skewer (20 cm long, 2 mm in diameter), and a vertically arranged bundle of natural grass (approximately 30 cm long). Substrates were replaced or cleaned with 70% ethanol and air-dried between trials to remove any residual silk. To analyze male silk probing behavior, we used six high-speed video recordings (500 fps, up to 8 s each) from a previous study^34^ that were also captured using the Miro LC320S high-speed camera. In each trial, a male was allowed to walk on and around dragline silk deposited by a different female in a transparent plastic box (17 × 8 × 6 cm), allowing us to observe and analyze their responses to female silk. During the prey capture experiments, we observed how spiders interacted with prey of different sizes, including smaller houseflies (*Musca domestica*) and larger house crickets (*Acheta domesticus*). Six males and six *P. mirabilis* females were filmed during prey capture (housefly and cricket), resulting in a total of 12 trials per sex (*N* = 6 spiders × 2 prey types). Each trial involved placing an individual spider in a transparent plastic box (17 × 8 × 6 cm), followed by the release of a live prey item into the enclosure. We included four video recordings from an earlier study (recorded at 1000 fps using a Photron Fastcam SA1.1 high-speed camera; Photron Inc., San Diego, CA, USA) to analyze prey capture involving crickets^64^. Two more recordings were done at 1000 fps per second using the Miro LC320S. For mating observations, six trials were conducted using six different male–female pairs. In each trial, an unmated female was placed in an open arena (40 × 30 × 4 cm) for 5 min to deposit dragline silk, which females typically lay down while roaming^35,37^. After this period, a male carrying a nuptial gift (a prey item wrapped in silk) in his chelicerae was introduced at the periphery of the arena. Upon encountering the female’s dragline silk, the male typically followed the silk trail toward the female, tapped her with his legs, and offered the nuptial gift. If the female accepted the gift and began feeding on it, the male proceeded to insert one of his pedipalps (sperm transfer organs) into the genital opening of the female (epigyne).

We analyzed all footage of the above-mentioned behavioral contexts in slow motion using ImageJ software (public domain, https://imagej.net/ij/). We noted which segments (tarsus, metatarsus, tibia, patella, and femur) of the leg or pedipalp made contact in these different contexts and which did not. The segments that contacted the substrates or partner at least once during a trial were considered contact areas for the specific appendages in that trial. We compared the number of trials in which contact occurred to the total number of trials (6–24 trials, depending on the context; see Supplementary Table 3) for all contexts, resulting in a contact probability ranging from 0–100%. The leg and pedipalp segments that made contact in more than 50% of the cases were labeled contact areas, whereas those below 50% were classified as infrequent contact areas. Those segments that did not contact the substrates during our trials were defined as non-contact areas.

### Statistics and reproducibility

Morphological differences in tip-pore sensilla shafts between sexes were compared using an unpaired, two-tailed Student’s *t*-test in Microsoft Excel (*N* = 30 sensilla per sex). Male olfactory choice data were compared against a random distribution using a binomial test in R (R Studio, version: 2024.12.0+467; *N* = 23 test replicates and *N* = 22 for double control). The significance threshold was set at *p* < 0.05 for all tests.

### Reporting summary

Further information on research design is available in the Nature Portfolio Reporting Summary linked to this article.

## Supplementary information

Supplementary Information

Description of Additional Supplementary Files

Supplementary Movie 1

Supplementary Movie 2

Supplementary Movie 3

Reporting summary

## Data Availability

The data are given in the main text and in the supplementary material. Further high-speed videos of contact and non-contact areas of body appendages experiment are available from the corresponding authors on reasonable request.
