# Supplementary material for: The chemosensory toolkit of the cursorial spider Pisaura mirabilis
Source: Commun Biol. 2025 Nov 29;8:1733. doi: 10.1038/s42003-025-09127-z (PMC12673132; doi:10.1038/s42003-025-09127-z)
Supplement: Supplementary file 1 — Supplementary Information [file 42003_2025_9127_MOESM1_ESM.pdf]

Supplementary information

The Chemosensory Toolkit of The Cursorial Spider *Pisaura mirabilis*

Mohammad Belal Talukder[1*], Carsten H. G. Müller[1], Andreas Fischer[1], Vedanti Mahimkar[1], Jonas O. Wolff[2], and Gabriele B. Uhl[1*]

[1]General and Systematic Zoology, University of Greifswald; Greifswald, 17489, Germany

[2]Evolutionary Biomechanics, University of Greifswald; Greifswald, 17489, Germany

*Corresponding authors: Email: gabriele.uhl@uni-greifswald.de, mb.talukder.bd@gmail.com

Supplementary Note 1: Touch versus no-touch area of body appendages

Using high-speed video recording, we analyzed the contact and non-contact regions of body appendages in *P. mirabilis* males and females during locomotion on various surfaces, prey capture, silk probing, and mating. During locomotion, the tarsus of all walking legs always contacted the substrate. The metatarsus occasionally contacted the substrate with frequencies ranging from 8% to 20%. The proximal leg segments, namely the tibia-patella and femur, did not contact the substrate during any of our locomotion trials (Supplementary Table 3). When males probed the dragline silk left behind by females, the tarsus of the 1st and 2nd walking legs, along with the cymbium (first segment of male's pedipalp) of the pedipalps, frequently contacted the silk (83%-100%). The tarsus of the 3rd and 4th walking legs occasionally contacted the silk (16%) (Supplementary Table 3). The rest of the segments, other than the tarsus of the walking legs, and the cymbium of pedipalps, did not contact the silk. During prey capture, both sexes frequently used the tarsus and metatarsus (contact frequencies: 58%-100%) to form a preybasket. The tibia was rarely in contact with the prey (1%-16%), whereas the femur did not contact the prey (Supplementary Table 3). During mating, the tarsus and metatarsus of the first three leg pairs in males and the first two leg pairs in females frequently contacted the mating partner (83%-100%). The tibia-patella occasionally contacted the mating partner of both sexes, with frequencies ranging from 16% to 33%, whereas the femur did not contact the mating partner in either sex (Supplementary Table 3). The pedipalps of both sexes were always in contact with the mating partner or during handling of the nuptial gift. We observed that in the walking legs, substrate contact occurs predominantly on the ventral and lateral surfaces, with little to no contact on the dorsal side. In contrast, substrate contact on the pedipalps is primarily limited to the dorsal and lateral surfaces.

Supplementary Figures

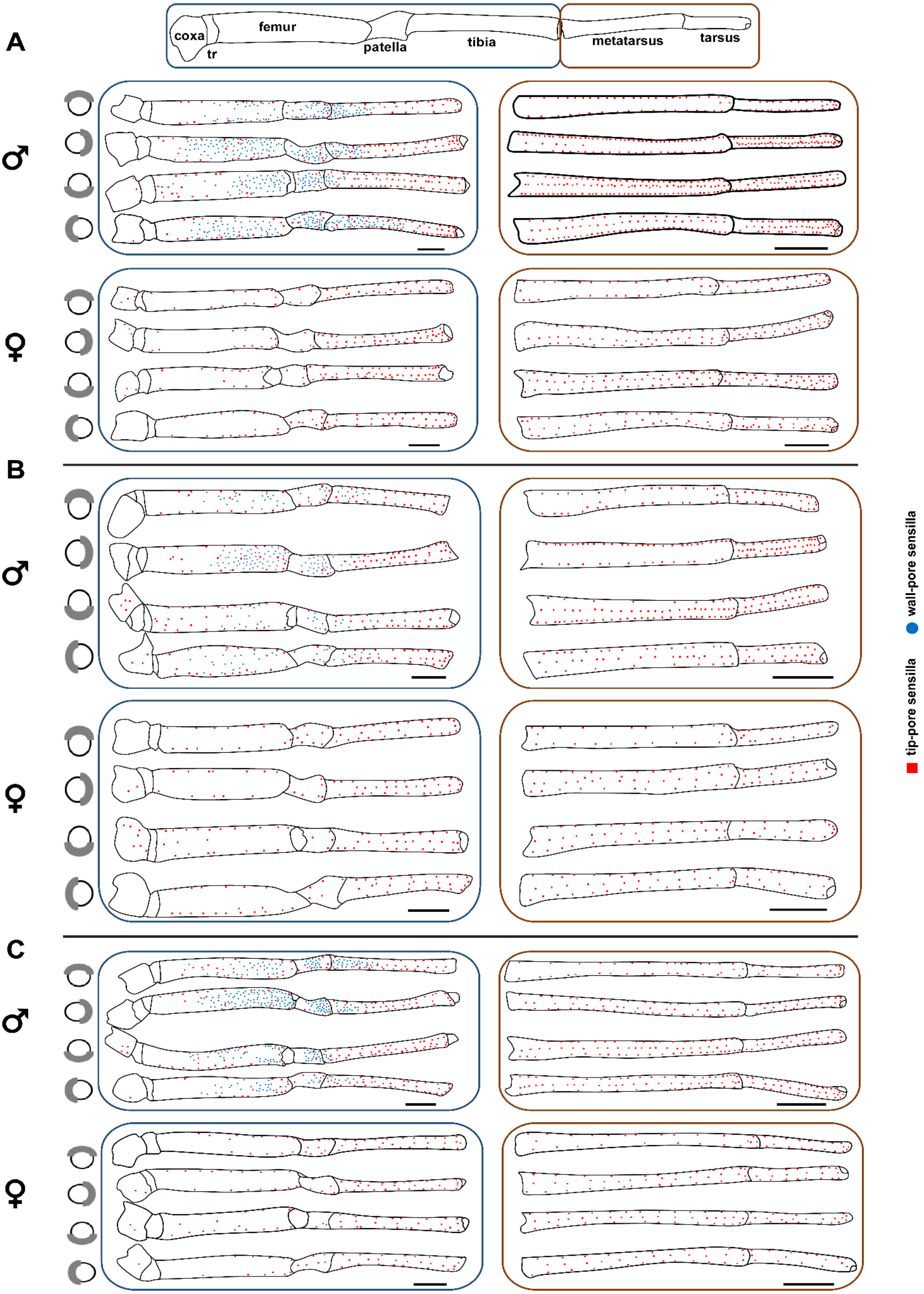

Supplementary Figure 1. Distribution of chemosensilla on the 2nd, 3rd, and 4th walking leg of *Pisaura mirabilis*. A 2nd walking leg of a male (♂) and a female (♀). **B** 3rd walking leg of a male (♂) and a female (♀). **C** 4th walking leg of a male (♂) and a female (♀). Red squares represent tip-pore sensilla and blue circles wall-pore sensilla. Each panel shows four perspectives from top to bottom: dorsal, prolateral, ventral, and retrolateral. Notably, wall-pore sensilla are present only in the males. Scale bars: 1 mm.

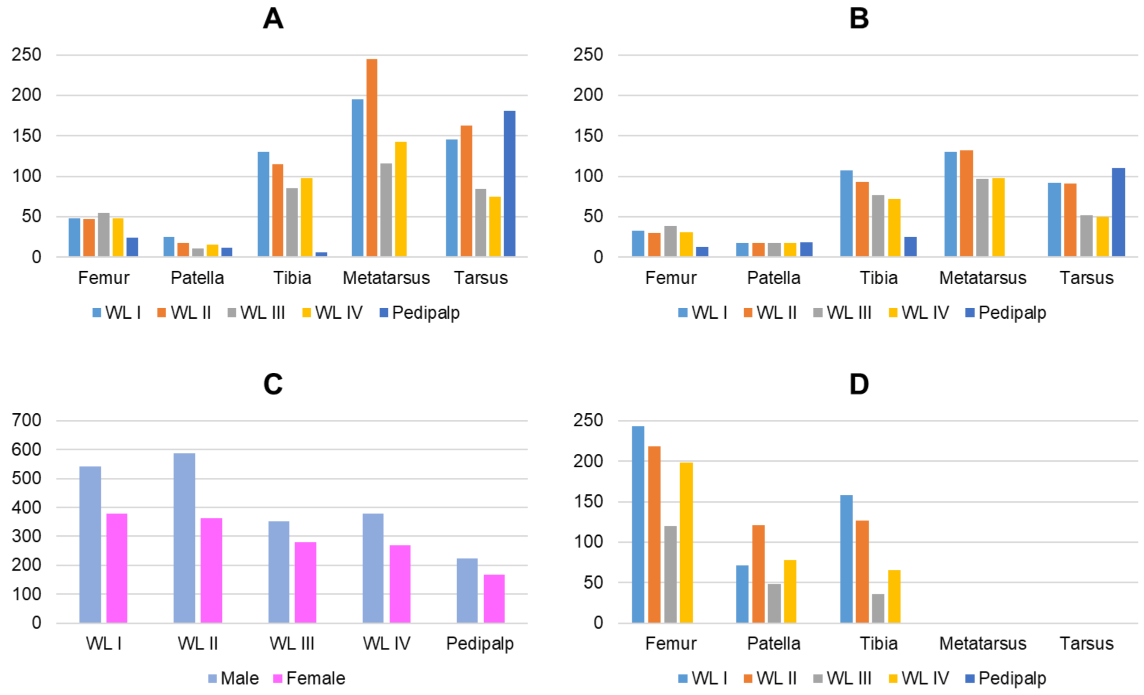

Supplementary Figure 2: Number and distribution of chemosensilla on the appendages of a male and a female *Pisaura mirabilis*. A Number and distribution of tip-pore sensilla on the appendages of a male and **B** a female. Note that there is no metatarsus in the pedipalp. **C** Comparison of the number of tip-pore sensilla on the appendages of a male and a female. **D** Number and distribution of wall-pore sensilla on the segments of walking legs of a male. Labels: WL I - WL IV represent the first to fourth walking legs. Note that wall-pore sensilla are absent on the pedipalp and none on metatarsus and tarsus.

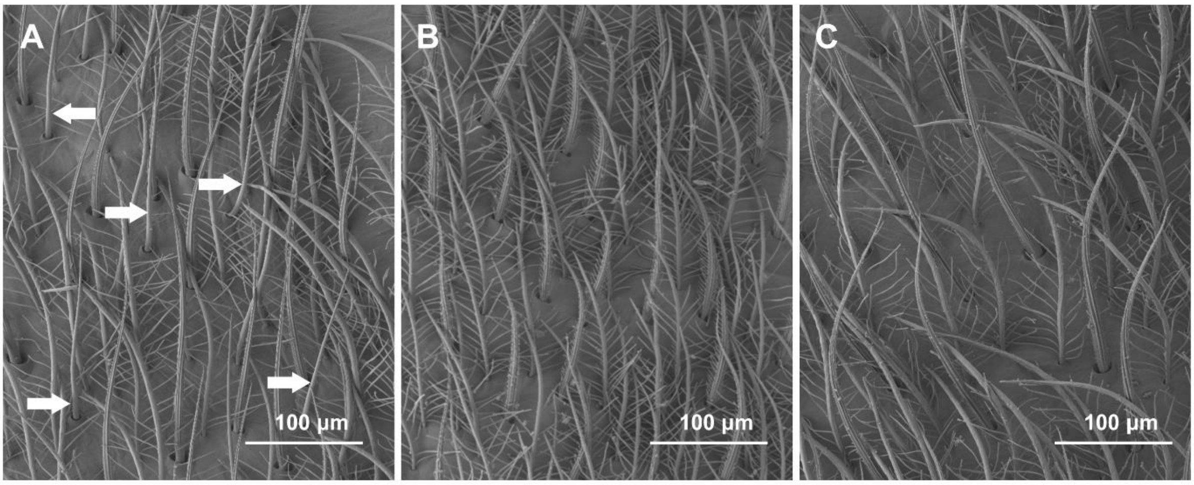

Supplementary Figure 3. Wall-pore sensilla occur exclusively in adult male *Pisaura mirabilis*. SEM overview of the femur region of the first walking leg of **A** an adult male, **B** a subadult male, and **C** an adult female. Five wall-pore sensilla are marked with white arrows on the femur of the adult male.

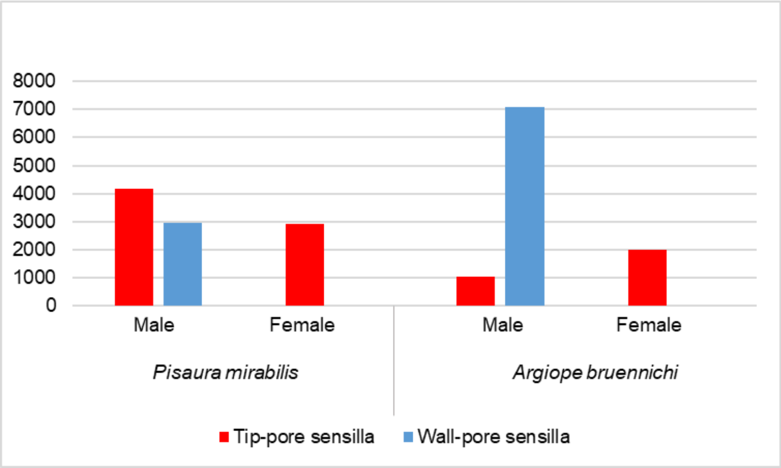

Supplementary Figure 4: **Comparison of total number of chemosensilla (tip-pore and wall-pore sensilla) between the cursorial spider *Pisaura mirabilis* and the orb-weaver *Argiope bruennichi*.** For tip-pore sensilla, one male and one female were examined per species. For wall-pore sensilla, one male was examined per species because these sensilla do not occur on females.

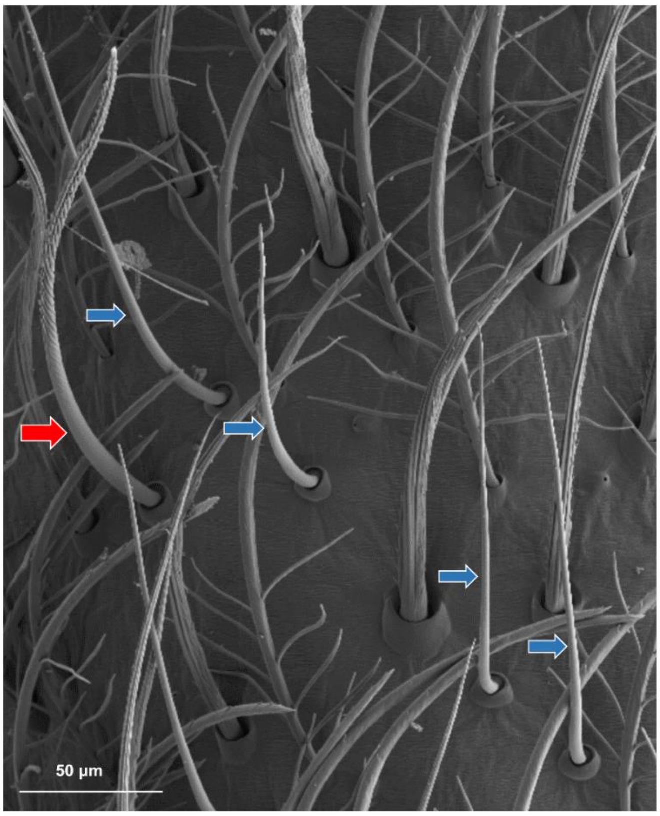

Supplementary Figure 5: Scanning electron microscopy overview of a tibia region on a walking leg of a male *Pisaura mirabilis*. A single tip-pore sensillum (red arrow) and four wall-pore sensilla (blue arrows) are visible.

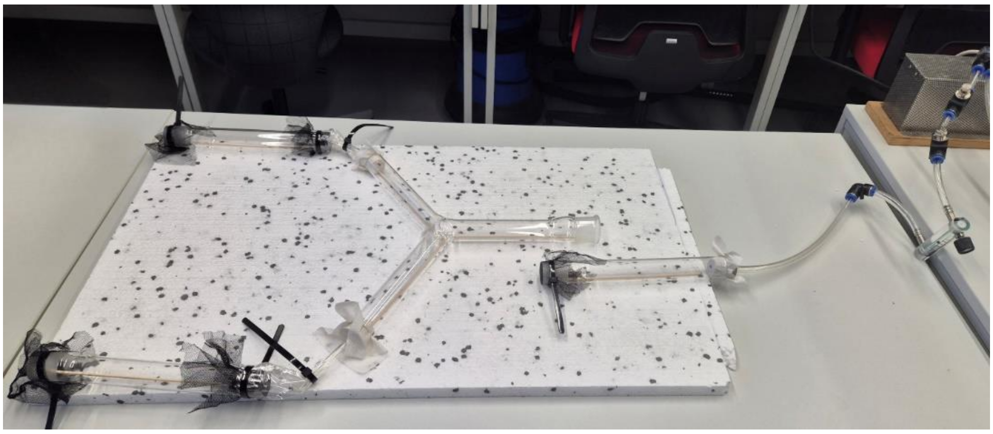

Supplementary Figure 6: Olfactometer used to test for attraction of male *Pisaura mirabilis* to female scent versus a stimulus-free control. Tubing connected each arm with stimulus chambers that held a female, or not. Males were introduced to a holding tube connected to a pump drawing 0.4 L/min air for two min prior connection to the Y-tube. A choice was considered when a male reached the end of a Y-arm. The whole setup is placed on Styrofoam to block any potential vibrational female signals.

Supplementary Tables

Leg segment	N	Sensillum length (µm)		Sensillum shaft diameter (µm)		Sensillum insertion angle (°)		Pore diameter (nm)	
		Male	Female	Male	Female	Male	Female	Male	Female
Femur	6	180.57±40.46	201.60±16.63	5.75±0.44	6.72±0.79	63.68±09.90	72.75±7.72	N=10 pores	N=10 pores
Patella	6	143.17±20.55	136.12±17.30	5.72±0.29	6.05±0.24	51.32±13.74	71.18±7.27		
Tibia	6	202.85±31.03	230.07±22.09	6.22±0.16	6.47±0.98	48.81±07.95	56.57±2.22		
Metatarsus	6	199.13±25.23	215.48±16.60	6.85±0.71	6.90±0.37	53.12±03.35	59.13±1.96		
Tarsus	6	150.05±07.75	157.45±19.33	6.68±0.37	6.75±0.49	49.05±07.48	46.27±5.54		
x̄±SD		175.15±37.39	188.14±40.80	6.24±0.65	6.58±0.71	53.20±10.83	61.18±11.45	599.1±150.26	561±106.06

Supplementary Table 1: Measurements of tip-pore sensilla on the segments of the 1st walking leg of *Pisaura mirabilis*. Values for each leg segment represent averages and standard deviations from six sensilla per sex. Pore diameters were measured from 10 randomly selected tip-pore sensilla distributed along the leg.

Leg segment	N	Sensilla length (µm)	Sensilla diameter (µm)	Pore diameter (nm)
Femur	5	148.62±09.94	2.18±0.10	42.60±1.85
Patella	5	136.26±07.51	2.08±0.23	40.20±2.56
Tibia	5	140.48±11.43	2.10±0.60	43.00±3.90
x̄±SD	15	141.79±11.41	2.12±0.16	41.93±3.26

Supplementary Table 2: Measurements of wall-pore sensilla on the segments of the 1st walking leg of a *Pisaura mirabilis* male. Values for each leg segment represent the averages and standard deviations based on five sensilla from a male. For average diameter of the sensillum pores, three pores from the five sensilla were measured.

		Male				Female			
		ta	mt	ti+pt	fe	ta	mt	ti+pt	fe
Locomotion	Leg I	100	25	0	0	100	12	0	0
	Leg II	100	20	0	0	100	8	0	0
	Leg III	100	8	0	0	100	8	0	0
	Leg IV	100	0	0	0	100	8	0	0
	pedipalp	62	n.a	0	0	79	n.a	8	0
Male probes female-produced dragline-silk	Leg I	100	0	0	0	-			
	Leg II	83	0	0	0				
	Leg III	16	0	0	0				
	Leg IV	16	0	0	0				
	pedipalp	83	n.a	0	0				
Prey capture	Leg I	100	100	8	0	100	83	16	0
	Leg II	100	91	8	0	91	58	16	0
	Leg III	100	91	0	0	83	58	8	0
	Leg IV	91	100	1	0	58	58	1	0
	pedipalp	100	n.a	0	0	100	n.a	0	0
Mating	Leg I	100	83	33	0	83	66	16	0
	Leg II	100	83	33	0	83	66	16	0
	Leg III	83	16	16	0	83	16	16	0
	Leg IV	33	0	0	0	16	16	0	0
	pedipalp	100	n.a	0	0	100	n.a	0	0

Supplementary Table 3: Probability (%) that body appendages of *Pisaura mirabilis* males and females come into contact with substrates. A During locomotion on 4 substrates (natural leaves, bundle of natural grass, flat surface, and horizontal skewer). Six males and six females were allowed to walk on all four substrates (N=6×4=24 trials for each sex). **B** During female-produced dragline-silk probing by males. Six males were exposed to dragline-silk left by females (N=6 trials). Note that female-produced dragline-silk probing trials were done only for male. **C** During prey capture. Six males and six females were filmed during prey (cricket and housefly) capturing (N=6×2 prey items =12 trials for each sex). **D** Contact with a mating partner during mating. Six couples were filmed immediately after their first touch with their mating partner (N=6 trials). In **A-D**: Data is specified for the segments (tarsus to femur) of the walking legs and the pedipalps (n.a. = lacks metatarsus). ta: tarsus, mt: metatarsus, ti: tibia, pt: patella, fe: femur.

	tarsus		metatarsus		tibia+patella		femur	
WL/Pod	male	female	male	female	male	female	male	female
Leg I	100	95	52	53	10	11	0	0
Leg II	96	92	53	44	10	11	0	0
Leg III	75	87	29	28	4	8	0	0
Leg IV	60	55	25	28	1	1	0	0
PP	86	90	n.a	n.a	0	3	0	0

Supplementary Table 4: Probability (%) that body appendages of *Pisaura mirabilis* males and females come into contact with substrates when all the contexts are pooled (locomotion, silk probing by male, prey capturing, and mating). We averaged of all percentage across all contexts; see table S3. For each sex, we analyzed 24 footages for locomotion in four different substrates, six footages for female-produced dragline-silk probing by male, 12 footages for two types of prey (cricket and housefly) capturing, and six footages of mating. Data is specified for the segments (tarsus to femur) of the walking legs and the pedipalps (n.a. = lacks metatarsus). WL: walking leg, Pod: podomeres, and PP: Pedipalp.
