# Supplementary material for: The chemosensory toolkit of the cursorial spider Pisaura mirabilis
Source: Commun Biol. 2025 Nov 29;8:1733. doi: 10.1038/s42003-025-09127-z (PMC12673132; doi:10.1038/s42003-025-09127-z)
Supplement: Supplementary file 2 — Description of Additional Supplementary Files [file 42003_2025_9127_MOESM2_ESM.docx]

**The Chemosensory Toolkit of the Cursorial Spider *Pisaura mirabilis***

**Authors:** Mohammad Belal Talukder^1*^, Carsten H. G. Müller^1^, Andreas Fischer^1^, Vedanti Mahimkar^1^, Jonas O. Wolff^2^, and Gabriele B. Uhl^1*^

**Affiliations:**

^1^General and Systematic Zoology, University of Greifswald; Greifswald, 17489, Germany

^2^Evolutionary Biomechanics, University of Greifswald; Greifswald, 17489, Germany

*Corresponding authors: Email: gabriele.uhl@uni-greifswald.de (G.B.U.), mb.talukder.bd@gmail.com (M.B.T.)

**Description of Additional Supplementary Files**

**File name:** Supplementary Movie 1

**Title:** Exemplary high-speed video recording of locomotion of *Pisaura mirabilis*.

**Description:** A female *Pisaura mirabilis* walking on fresh common nettles (a plant on which the species is frequently found in the field). Video playback rate adjusted to 100 frames per second, representing a 5x slow-motion effect from the original recording speed of 500 frames per second.

**File name:** Supplementary Movie 2

**Title:** Exemplary high-speed video recording of prey capturing of *Pisaura mirabilis*.

**Description:** A female *Pisaura mirabilis* capturing prey (house crickets; Acheta domesticus). Video playback rate adjusted to 100 frames per second, representing a 5x slow-motion effect from the original recording speed of 500 frames per second.

**File name:** Supplementary Movie 3

**Title:** Exemplary high-speed video recording of a mating sequence of *Pisaura mirabilis*.

**Description:** Video playback rate adjusted to 100 frames per second, representing a 5x slow-motion effect from the original recording speed of 500 frames per second.
