# Supplementary material for: The chemosensory toolkit of the cursorial spider Pisaura mirabilis
Source: Commun Biol. 2025 Nov 29;8:1733. doi: 10.1038/s42003-025-09127-z (PMC12673132; doi:10.1038/s42003-025-09127-z)
Supplement: Supplementary file 6 — Reporting summary [file 42003_2025_9127_MOESM6_ESM.pdf]

nature portfolio

Corresponding author(s): Gabriele B. Uhl, Mohammad Belal Talukder

Last updated by author(s): 2025.10.10

Reporting Summary

Nature Portfolio wishes to improve the reproducibility of the work that we publish. This form provides structure for consistency and transparency in reporting. For further information on Nature Portfolio policies, see our Editorial Policies and the Editorial Policy Checklist.

Statistics

For all statistical analyses, confirm that the following items are present in the figure legend, table legend, main text, or Methods section.

n/a	Confirmed	
☐	☒	The exact sample size (*n*) for each experimental group/condition, given as a discrete number and unit of measurement
☐	☒	A statement on whether measurements were taken from distinct samples or whether the same sample was measured repeatedly
☐	☒	The statistical test(s) used AND whether they are one- or two-sided<br>*Only common tests should be described solely by name; describe more complex techniques in the Methods section.*
☒	☐	A description of all covariates tested
☒	☐	A description of any assumptions or corrections, such as tests of normality and adjustment for multiple comparisons
☐	☒	A full description of the statistical parameters including central tendency (e.g. means) or other basic estimates (e.g. regression coefficient) AND variation (e.g. standard deviation) or associated estimates of uncertainty (e.g. confidence intervals)
☒	☐	For null hypothesis testing, the test statistic (e.g. *F*, *t*, *r*) with confidence intervals, effect sizes, degrees of freedom and *P* value noted<br>*Give P values as exact values whenever suitable.*
☒	☐	For Bayesian analysis, information on the choice of priors and Markov chain Monte Carlo settings
☒	☐	For hierarchical and complex designs, identification of the appropriate level for tests and full reporting of outcomes
☒	☐	Estimates of effect sizes (e.g. Cohen's *d*, Pearson's *r*), indicating how they were calculated

Our web collection on statistics for biologists contains articles on many of the points above.

Software and code

Policy information about availability of computer code

Data collection	NA
Data analysis	In the current study, male olfactory choice data were statistically compared against binomial or Fisher's exact tests in R. The high-speed video footages of contact and non-contact areas of body appendages experiment were analyzed using Image J.

For manuscripts utilizing custom algorithms or software that are central to the research but not yet described in published literature, software must be made available to editors and reviewers. We strongly encourage code deposition in a community repository (e.g. GitHub). See the Nature Portfolio guidelines for submitting code & software for further information.

Data

Policy information about availability of data

All manuscripts must include a data availability statement. This statement should provide the following information, where applicable:

- Accession codes, unique identifiers, or web links for publicly available datasets
- A description of any restrictions on data availability
- For clinical datasets or third party data, please ensure that the statement adheres to our policy

The data are given in the main text and in the supplementary material. Repetitive high-speed videos of contact and non-contact areas of body appendages experiment are available from the corresponding authors on reasonable request.

Research involving human participants, their data, or biological material

Policy information about studies with human participants or human data. See also policy information about sex, gender (identity/presentation), and sexual orientation and race, ethnicity and racism.

Reporting on sex and gender	NA
Reporting on race, ethnicity, or other socially relevant groupings	NA
Population characteristics	NA
Recruitment	NA
Ethics oversight	NA

Note that full information on the approval of the study protocol must also be provided in the manuscript.

Field-specific reporting

Please select the one below that is the best fit for your research. If you are not sure, read the appropriate sections before making your selection.

☐ Life sciences ☐ Behavioural & social sciences ☒ Ecological, evolutionary & environmental sciences

For a reference copy of the document with all sections, see nature.com/documents/nr-reporting-summary-flat.pdf

Ecological, evolutionary & environmental sciences study design

All studies must disclose on these points even when the disclosure is negative.

Study description	This study investigates the chemosensory toolkit of a cursorial spider by combining detailed morphological analyses with behavioral assays. We examined sensory organs using high-resolution imaging techniques and quantified behavioral responses through olfactometer tests (23 replicates) and contact vs. non-contact experiments (6 replicates per context).
Research sample	This study was conducted on the cursorial spider species Pisaura mirabilis, a well-studied representative of cursorial hunting spiders. Pisaura mirabilis belongs to the class Arachnida and the order Araneae in the animal kingdom. Subadult and adult males and females were used in the experiments, with specimens collected from various districts in northern Germany.
Sampling strategy	Spiders were collected randomly from the field. Sensory organs were examined using high-resolution imaging techniques, with 1–4 individuals used for morphological analyses, which provided sufficient detail for comparison. Behavioral responses were quantified using 23 replicates in olfactometer tests and 6 replicates per context in contact versus non-contact experiments.
Data collection	M.B.T., C.H.G.M. collected the morphological data obtained using scanning and transmission electron microscopy. M.B.T., A. F., and V.M. collected behavioral studies data using high-speed video recording and Y-tube olfactometer test.
Timing and spatial scale	Juvenile, subadult, and adult P. mirabilis of both sexes were collected each year from 2021-2024 from grasslands in northern Germany.
Data exclusions	No data were excluded from this study
Reproducibility	The experimental findings are reproducible and were successfully validated through repeated trials.
Randomization	We selected individual spiders at random for each part of the study to avoid sampling bias and ensure representative results.
Blinding	Blinding was not applicable to our study, as measures were taken to minimize observer bias through standardized procedures and objective data collection.

Did the study involve field work? ☐ Yes ☒ No

Reporting for specific materials, systems and methods

We require information from authors about some types of materials, experimental systems and methods used in many studies. Here, indicate whether each material, system or method listed is relevant to your study. If you are not sure if a list item applies to your research, read the appropriate section before selecting a response.

Materials & experimental systems

n/a	Involved in the study
☒	☐ Antibodies
☒	☐ Eukaryotic cell lines
☒	☐ Palaeontology and archaeology
☐	☒ Animals and other organisms
☒	☐ Clinical data
☒	☐ Dual use research of concern
☒	☐ Plants

Methods

n/a	Involved in the study
☒	☐ ChIP-seq
☒	☐ Flow cytometry
☒	☐ MRI-based neuroimaging

Animals and other research organisms

Policy information about studies involving animals; ARRIVE guidelines recommended for reporting animal research, and Sex and Gender in Research

Laboratory animals	This study did not involve laboratory animals.
Wild animals	Juvenile, subadult, and adult Pisaura mirabilis (Clerck, 1757) individuals of both sexes were collected each year from 2021-2024 from grasslands in Greifswald, Germany and brought to the laboratory. The spiders were individually collected in Drosophila culture tubes and brought to the laboratory. After the study spiders were fixed in 70% ethanol and stored.
Reporting on sex	Experiments were conducted on both sexes, and sex was included as a factor in the study design. Sex determination was based on genital morphology: males were identified by the presence of modified pedipalps, which serve as sperm transfer organs
Field-collected samples	Juvenile, subadult (one molt prior to adulthood), and adult Pisaura mirabilis (Clerck, 1757) individuals of both sexes were collected each year from 2021-2024 from grasslands in Greifswald, Germany (54° 05' 49.91''N 13° 23' 16.58'' E) and brought to the laboratory. The spiders were individually housed in Drosophila culture tubes measuring 5 cm in diameter and 10 cm in height, with a netted top and a sponge lid at the bottom35. Inside each vial, a substrate of artificial aquarium plants was provided. To maintain high humidity levels, vials with sponges at the bottom were placed in trays filled with water. The spiders were kept at a constant temperature of 22°C (±5°C) under a 12:12 h light–12 h dark cycle. Adult and subadult spiders were fed one housefly (Musca domestica) or blowfly (Lucilia caesar) twice a week, whereas juveniles were provided with several fruit flies (Drosophila hydei) on the same schedule.
Ethics oversight	Ethical approval was not required for this study, as all experiments were non-invasive behavioral assays and were conducted in accordance with best practices to minimize handling stress and ensure animal welfare.

Note that full information on the approval of the study protocol must also be provided in the manuscript.

Plants

Seed stocks	NA
Novel plant genotypes	NA
Authentication	NA
